# End‐to‐end deep learning pipeline for automated IMRT planning in lymphoma: Feasibility and limitations

**DOI:** 10.1002/acm2.70702

**Published:** 2026-08-04

**Authors:** Denis Kutnár, Ivan Richter Vogelius, Hristo Atanasov Georgiev, Katrin Elisabet Håkansson, Lena Specht, Jens Petersen

**Affiliations:** ^1^ Department of Computer Science University of Copenhagen Copenhagen Denmark; ^2^ Department of Oncology Rigshospitalet Copenhagen Denmark; ^3^ Faculty of Health and Medical Sciences University of Copenhagen Copenhagen Denmark

**Keywords:** automated treatment planning, deep learning, fluence map, IMRT, lymphoma, radiotherapy

## Abstract

**Background:**

Intensity‐modulated radiation therapy (IMRT) is widely used for lymphoma because a multileaf collimator (MLC) can sculpt dose around irregular targets. Planning, however, is time‐consuming and difficult to standardize. Fluence map prediction (FMP) using deep learning (DL) could potentially be used to automate planning, but it has not been evaluated in full‐body lymphoma, where prescriptions, organ‐at‐risk (OAR) sets, and anatomical presentations vary substantially. Here, we evaluate multiple DL configurations to determine whether they can be adapted to lymphoma and generate IMRT plans that are deliverable in a clinical treatment planning system (TPS).

**Purpose:**

To investigate the feasibility and limitations of DL–based dose and fluence prediction methods in the heterogeneous setting of lymphoma.

**Methods:**

A single‐institution lymphoma cohort (571 patients, 619 plans) was replanned from clinical volumetric modulated arc therapy (VMAT) to a consistent 15‐field IMRT reference using the Eclipse Scripting API (ESAPI), yielding 9285 beam‐level pairs. We implemented a two‐stage DL pipeline that first predicts beam‐wise dose from CT and planning target volume (PTV), then maps dose to fluence, and finally computes the corresponding dose in the TPS after leaf sequencing. We evaluated several architectural and training variants, from replication‐focused models to configurations with a clinical loss based on dose–volume histogram (DVH) aimed at moving beyond simple plan replication. For each configuration, we analysed how these choices influenced predicted dose realism and the quality of the resulting plans.

**Results:**

In Stage 1 (dose prediction), replication‐focused DL models were numerically inferior to the IMRT and VMAT references, while DVH‐driven variants yielded superior target DVHs. After fluence mapping and TPS sequencing, all configurations produced machine‐deliverable IMRT plans but with lower target coverage and higher hot spots than the references; fluence was largely preserved by MLC sequencing, indicating that current limitations mainly stem from the predicted dose distributions and the learned dose‐to‐fluence mapping.

**Conclusions:**

DL–based dose and fluence prediction can generate fast, machine‐deliverable IMRT plans for heterogeneous lymphoma that lie in the clinical ballpark, but they remain dosimetrically inferior to manual IMRT and VMAT, highlighting the current limitations of these methods for automated planning in this setting.

## 1 INTRODUCTION

Radiotherapy (RT) remains a cornerstone of lymphoma management, providing durable local control while limiting dose to normal tissues. Intensity‐modulated radiation therapy (IMRT) is widely used because a multileaf‐collimator (MLC) can sculpt dose around the irregular volumes characteristic of Hodgkin and many nodal non‐Hodgkin presentations.^1^, ^2^, ^3^ Once contours are approved, a senior planner typically spends hours iteratively adjusting objective weights and normal‐tissue trade‐offs before the final dose–volume review.^4^, ^5^, ^6^ Variation in planner experience translates into clinically meaningful differences in conformity, homogeneity, and organs‐at‐risk (OAR) sparing.^7^

Planning complexity is amplified in lymphoma. Targets can arise anywhere in the body, both within and outside the lymphatic system, and prescription schedules span from approximately 20 Gy consolidation after complete metabolic response to 40–45 Gy boosts for gross or residual disease.^1^, ^2^, ^3^, ^8^ This wide variation in prescription dose and target location precludes meaningful use of QUANTEC^9^ style dose constraints for normal tissues in lymphoma, leaving a clinical need for a patient‐specific assessment of plan quality. Commercial knowledge‐based planning models are typically commissioned for a single site with fixed OAR sets and beam geometry; those assumptions are rarely satisfied across the heterogeneous lymphoma spectrum.^10^, ^11^ A site‐agnostic, fully automatic IMRT workflow that generalises across presentations and fractionation remains an open challenge.

Deep learning (DL) methods can now infer high‐quality 3D dose distributions directly from the planning computed tomography (CT) and structure set, with promising results reported for several anatomical sites.^12^, ^13^, ^14^ However, aggregate‐dose predictors bypass the beam‐specific physics of attenuation and MLC sequencing that determine deliverability. Fluence map prediction (FMP) addresses this by estimating field‐level intensity modulation that can be directly sequenced, and early studies in nasopharyngeal, pancreas, prostate, and head‐and‐neck cancers demonstrate rapid generation of deliverable fluence,^15^, ^16^, ^17^, ^18^ typically in well‐curated, single‐site cohorts with fixed prescriptions/fractionations and standardised OAR sets. Most existing FMP frameworks are trained to replicate a single clinical reference plan per patient, even though inverse planning is inherently multi‐objective, and many Pareto‐optimal solutions can exist for a given case.^19^ In heterogeneous diseases such as lymphoma, with widely varying prescriptions, target locations, and OAR definitions, it is unclear how well such dose‐ and fluence‐prediction approaches generalise, or whether they can reliably produce machine‐deliverable plans of acceptable quality in a clinical treatment planning system (TPS).

In this work, we evaluate an end‐to‐end DL framework for lymphoma IMRT that predicts beam‐wise dose, converts predicted dose to fluence, and generates machine‐deliverable plans through a clinical TPS. Using a large, heterogeneous single‐institution cohort, we assess multiple architectural and training configurations and compare both intermediate dose predictions and fully deliverable plans against consistent IMRT and VMAT references. Our goal is not to demonstrate clinical superiority, but to characterise the feasibility and limitations of DL–based dose and fluence prediction in the heterogeneous setting of full‐body lymphoma.

## 2 METHODS

### 2.1 Patient cohort

This single‐institution, retrospective study was approved by the Ethics Committee. From the oncology information system (ARIA v18.0, Varian Medical Systems), we retrieved 619 VMAT plans from 571 consecutive lymphoma patients treated between 2009 and 2021, including 97 palliative plans and 522 curative‐intent plans. All DICOM RT objects (CT, RTPLAN, RTSTRUCT, RTDOSE) were fully anonymised prior to analysis. Per the approved protocol, the clinical data were used on‐site only and cannot be shared externally. The cohort spans the full body, with substantial dosimetric diversity; planning target volume (PTV) ranged from 5.4 to 2282 cm^3^, and prescriptions from 4 to 60 Gy (Table 1).

**TABLE 1:** Patient demographics and plan characteristics for the study cohort.

Characteristic	Value
**Cohort size**	
Patients, *n*	571
Treatment plans, *n*	619
Palliative plan intent, *n*	97
Curative plan intent, *n*	522
Plans per patient (median, range)	1 (1–3)
**Sex**	
Male, *n*	334
Female, *n*	237
**Age (years)**	
Mean ± SD	69 ± 16 years
Median (range)	73 (26–103 years)
**PTV volume (cm** 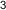 **)**	
Mean ± SD	294 ± 306 cm^3^
Median (range)	195 cm^3^ (5.4–2,282 cm^3^)

Abbreviations: PTV, planning target volume; SD, standard deviation.

### 2.2 Clinical baseline: VMAT‐to‐IMRT re‐planning

All treatments were delivered with VMAT; photon dose was computed using Acuros XB (*n* = 326/619) or the Anisotropic Analytical Algorithm (AAA; n = 293/619). To accommodate the anatomical and prescription heterogeneity of lymphoma within a consistent beam‐wise learning framework, the clinical VMAT plans were re‐planned to a standardised 15‐field IMRT geometry. The 15‐field arrangement provides broad angular coverage and substantial fluence‐modulation capacity, making it a reasonable fixed‐field approximation of arc‐based delivery for complex planning cases.^20^, ^21^ This representation enables DL‐predicted fluence maps to be imported into the clinical TPS, converted into deliverable MLC motion through leaf sequencing, and recalculated to generate machine‐deliverable treatment plans, allowing end‐to‐end evaluation of clinically relevant output.To construct this reference, we implemented an ESAPI v18.0 script in a research instance of Eclipse TPS (Varian Medical Systems, Palo Alto, CA) that: (1) transfers CT and RTSTRUCT to the research workspace; (2) copies all VMAT optimization objectives, priorities, and weights for targets and normal tissues; (3) creates a fixed set of 15 equi‐angular coplanar 6 MV photon beams (100 cm source‐to‐axis distance, HD‐120 MLC) for every case, regardless of the original VMAT arc setup; (4) performs sliding‐window IMRT optimization with automatic leaf sequencing using the copied objectives; and (5) recalculates dose with Acuros XB. Each VMAT‐to‐IMRT conversion ran fully automatically in <5 min per case and defines the IMRT baseline used as a consistent reference for evaluating the DL–generated plans.

### 2.3 Data preprocessing and dataset splits

All DICOM RT objects (CT, RTSTRUCT, RTDOSE, and field fluence maps) were exported with ESAPI and processed in Python. Each modality was rigidly realigned to the treatment isocenter and resampled to an isotropic 2.5 mm grid (B‐spline interpolation for CT, trilinear for dose, nearest‐neighbour for masks).

A custom 3D beam's eye view (BEV) transform (100 cm source‐to‐axis distance) re‐projected each field‐dose and the corresponding CT into beam coordinates using a standard cone‐beam geometry: the central‐axis slice defined the reference plane, and peripheral voxels were mapped along divergent rays to account for geometric divergence. Volumes were then cropped in the axial plane (perpendicular to the beam axis) using the recorded MLC Y1/Y2 jaw positions plus a 50 mm margin, removing anatomy non‐essential for dose prediction or calculation. To ensure uniform tensor size, all channels were symmetrically padded or cropped to a canonical 128×256×256 shape. Field doses were stored in relative units (Gy divided by the plan's target prescription dose), and channel‐specific training‐set means (μ) and standard deviations (σ) were used to *z*‐score‐normalise CT, field doses, and fluence maps.

Our dataset included a wide variety of plans, including cases with multiple dose levels, multiple PTVs, and multiple PTV‐related optimisation structures, underlining the complexity of lymphoma planning. To simplify the learning objective, we split the 619 plans into two groups: 317 plans with multiple dose levels or PTV constraints, and 302 plans with a single prescription level, which we treated as the main dataset. The multi‐prescription plans were used only for a pre‐training phase (split in 70/30 ratio for training and validation)^22^ to expose the networks to a broader range of dose patterns, which we considered sufficient for this purpose. The main single‐prescription dataset was then split into a training set (195 plans), a validation set (46 plans), and a held‐out test set (61 plans) at a 65/15/20 ratio. The train/validation/test splits were performed at the patient level to prevent data leakage between subsets and ensure a patient‐independent held‐out evaluation.

### 2.4 DL framework for lymphoma IMRT

**FIGURE 1:**
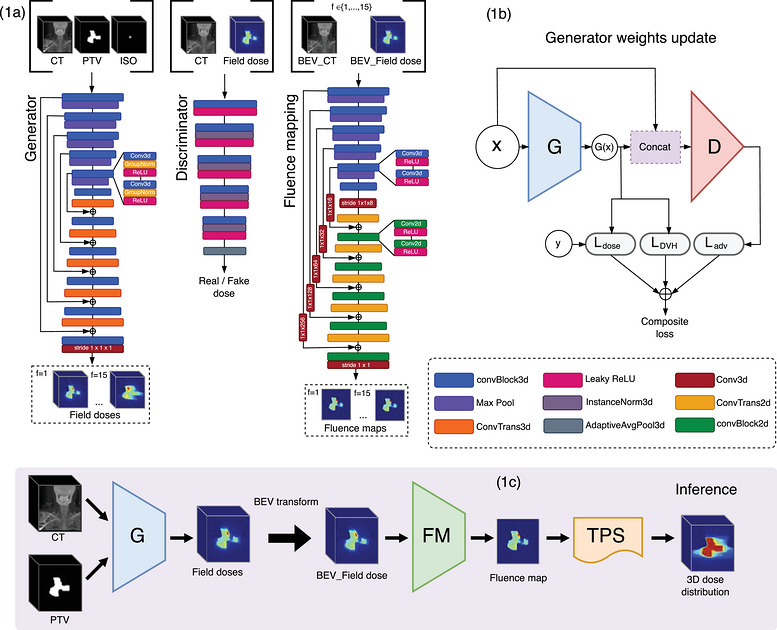
Framework overview. (a) Network architectures for the generator (G), discriminator (D), and fluence mapping (FM) modules. (b) Training schematic with the loss terms used to update G. The input *x* consists of computed tomography (CT), the planning target volume (PTV), and the treatment isocenter (ISO). The output G(*x*) is a set of 15 beam‐wise 3D field doses; the corresponding treatment‐planning‐system (TPS) dose is denoted *y*. The legend shows color‐coded network layers; building blocks include convBlock3d and convBlock2d. Red Conv3d indicates anisotropic‐stride convolutions. (c) Inference schematic using the beam's‐eye‐view (BEV) transformation.

We designed an end‐to‐end DL framework for lymphoma IMRT with three stages (Figure 1). Stage 1 predicts beam‐wise 3D dose from the CT and PTV. Stage 2 converts each predicted field dose to a 2D fluence map. Stage 3 imports the predicted fluence into Eclipse via ESAPI, performs MLC sequencing, and computes the final dose in the TPS. We chose this dose‐to‐fluence design to keep the intermediate dose closely tied to clinically interpretable dose objectives and beam‐level physics. Previous work has shown that combining CT and dose yields promising results for fluence prediction.^23^ We decided to investigate this further and train our fluence predictor on paired images of BEV‐transformed CT scans and dose distributions predicted in Stage 1. In this study we focus on evaluating how such an end‐to‐end approach behaves in the heterogeneous setting of full‐body lymphoma.

### 2.5 Stage 1: Beam‐wise dose prediction

Stage 1 predicts a stack of 15 beam‐wise 3D dose from CT and PTV prior to BEV transformation, providing the planning layer of the pipeline by approximating field‐dose physics and the dose apportionment across beams. All configurations C1–C6 (Table 2, further explained in Section 2.8) use a five‐level 3D U‐Net generator (channels 64‐1024) with options for adversarial training, an isocenter channel, pre‐training on multi‐prescription plans, a clinical dose–volume histogram (DVH) term, and different voxel resolutions. Inputs to the generator were: (i) CT (Hounsfield units), (ii) a binary PTV mask, and (iii) an optional isocenter sphere (3 cm radius, centered at the treatment isocenter; used in C3–C6). In C2–C6, a 3D ResNet discriminator (five residual blocks) operates on CT and dose only, independent of the PTV, to encourage physically plausible dose distributions. The generator minimizes:

$$L=L_{\text{dose}}+\lambda \;L_{\text{adv}}+\gamma \;L_{\mathrm{DVH}}$$

**TABLE 2:** Overview of experimental configurations used for dose‐prediction model development.

Config	Pre‐training phase	GAN discriminator	ISO‐blob input	Clinical DVH loss	Dose grid
C1	✗	✗	✗	✗	5.0 mm
C2	✗	✓	✗	✗	5.0 mm
C3	✗	✓	✓	✗	5.0 mm
C4	✓	✓	✗	✗	5.0 mm
C5	✓	✓	✓	✓	5.0 mm
C6	✓	✓	✓	✓	2.5 mm

*Note*:Each configuration combines different training strategies and architectural additions, including a GAN discriminator, an ISO input channel, clinical DVH loss, and the dose‐grid resolution.

Abbreviations: DVH, dose–volume histogram; GAN, generative adversarial network; ISO, isocenter.

For generator G and discriminator D, the adversarial term was defined using a standard GAN objective. The discriminator loss was

$$L_{D}=\frac{1}{2}\left(-E_{\left(x,y\right)}\left[\log D(x,y)\right]-E_{x}\left[\log\left(1-D(x,G(x))\right)\right]\right),$$

where x denotes the conditioning input and y the reference beam dose. The adversarial term used in the generator objective was

$$L_{\text{adv}}=-E_{x}\left[\log D(x,G(x))\right].$$

For configurations C5 and C6, the DVH‐based term was computed on the summed 3D dose,

$$\hat{d}=\sum_{f=1}^{15}G{\left(x\right)}_{f},$$

and defined as

$$L_{\mathrm{DVH}}\left(\hat{d}\right)=L_{D_{98}}\left(\hat{d}\right)+L_{D_{2}}\left(\hat{d}\right)+L_{D_{\max}}\left(\hat{d}\right)+L_{D_{\mathrm{mean}}}\left(\hat{d}\right),$$

where each term is a squared penalty on the deviation of the corresponding DVH metric from its desired clinical target, evaluated within the PTV.

In the initial supervised phase, we optimize $L_{\text{dose}}$ (voxel‐wise mean squared error, MSE, to the reference dose) to learn the core dose‐deposition structure; $L_{\text{adv}}$ and $L_{\mathrm{DVH}}$ are then gradually introduced to encourage physically plausible dose patterns and convergence toward clinically reasonable DVH endpoints. Specifically, $L_{\mathrm{DVH}}$ penalizes deviations from $D_{98\%}\;\geq 98\%$, $D_{2\%}\;\leq 102\%$, $D_{\max}\;\leq 105\%$, and $D_{\text{mean}}\;\approx 100\%$ of the prescription. λ is linearly ramped from 0 to 0.20 over 2000 epochs, after which γ is introduced during fine‐tuning (C5 to C6) and ramped from 0 to 1. This staged schedule first stabilizes spatial dose features, then refines DVH endpoints.

Networks were optimized with Adam ($\beta_{1}=0.9$, $\beta_{2}=0.999$) using an initial learning rate of $1\times 10^{-4}$ (found via LR‐finder) and a one‐cycle annealing schedule. The batch size was six, with generator and discriminator updated in a 1:1 ratio. Early stopping was triggered if none of $\left\{D_{98\%},\;D_{2\%},\;D_{\max},\;D_{\text{mean}}\right\}$ improved on the validation set for 30 epochs, or if train and validation MSE diverged by more than an order of magnitude. The discriminator's binary cross‐entropy (BCE) was monitored to keep the adversarial game balanced (with a combined BCE in the range 0.2–0.8).

### 2.6 Stage 2: FMP

Serving as a physics‐inversion layer, the second network maps the predicted beam‐wise dose volumes into 2D fluence maps, which are then passed to the clinical leaf sequencer in Stage 3. A hybrid 3D/2D U‐Net is employed: five 3D downsampling blocks encode the BEV‐transformed CT together with the stack of BEV‐transformed predicted field doses for all 15 beams. At the bottleneck, the feature tensor is collapsed along the beam axis, after which a 2D decoder reconstructs a multi‐channel fluence output (15 fluence maps; canonical size 128×256). Skip connections are similarly collapsed to preserve alignment between encoder and decoder features. Predicting all beam fluence maps jointly at the plan level allows shared features to capture interactions between beams, rather than treating each fluence map in isolation.^23^, ^24^, ^25^, ^26^ The model is trained using the same optimiser settings as Stage 1 (Adam, $\beta_{1}=0.9$, $\beta_{2}=0.999$; initial learning rate $1\times 10^{-4}$, one‐cycle schedule) with a batch size of six. Training minimises MSE between predicted and reference fluence and typically converges within 40 epochs, with early stopping triggered after 20 epochs without validation improvement.

### 2.7 TPS integration and dose calculation

A single ESAPI v18.0 script converts the network output into a fully sequenced IMRT plan deliverable on a Varian TrueBeam linac with an HD‐120 MLC. The predicted fluence maps (.optimal_fluence) are imported into Eclipse, registered to the TPS isocenter, and mapped to the beam‐limiting device coordinate system. The script then calls the clinical sliding‐window leaf sequencer (Varian Leaf Motion Calculator v18.0.0) for an HD‐120 MLC at 6 MV and triggers final dose calculation with Acuros XB v18.0.0, yielding a machine‐deliverable plan.

### 2.8 Experimental design

Six configurations (C1–C6, Table 2) were defined to probe the impact of key Stage 1 design choices while keeping Stages 2 and 3 fixed. All configurations share the same 3D U‐Net backbone for beam‐wise dose prediction and the same dose‐to‐fluence network and TPS sequencing; they differ only in adversarial training, the isocenter channel, pre‐training on multi‐prescription data, a DVH‐based clinical term, and voxel‐grid resolution.

C1–C4 are replication‐focused variants that use voxel‐wise MSE to match the IMRT reference dose. C1 is a basic supervised baseline. C2 adds an adversarial discriminator to test whether adversarial training can regularise spatial dose structure beyond MSE alone.^27^, ^28^ C3 augments the inputs with an isocenter channel; given that lymphoma targets can occur anywhere in the body, we hypothesised that explicitly encoding the isocenter might help the network represent beam depth and geometry relative to the PTV across different anatomical sites.^29^, ^30^, ^31^ C4 introduces pre‐training on multi‐prescription cases before fine‐tuning on single‐prescription plans, allowing the model to first see a broader range of dose patterns and then adapt to the single‐prescription setting.^32^, ^33^, ^34^

C5 and C6 build on C4 by adding a DVH‐based clinical term and, in C6, increasing the voxel resolution. The DVH term supplements the MSE loss with penalties on target $D_{98\%}$, $D_{2\%}$, $D_{\max}$, and $D_{\text{mean}}$ to more directly reflect clinical objectives. This is motivated by the multi‐objective nature of inverse planning: many Pareto‐optimal solutions can exist for a given case, and pure replication of a single reference plan may not capture clinically acceptable alternatives with different trade‐offs.^35^, ^36^ C6 further increases the voxel‐grid resolution to match the native 2.5 mm resolution at which the fluence maps are defined in the TPS, to test whether retaining this spatial detail in the dose prediction influences downstream plan quality.

For each configuration, the pipeline was run end‐to‐end: beam‐wise dose prediction, dose‐to‐fluence inversion, and TPS‐based MLC sequencing and dose calculation. The best‐performing setup on the validation cohort was repeated with three random seeds to assess stochastic variability of training due to random initialization of neural network weights and minibatch shuffling; the remaining configurations were run once, which we deemed sufficient for ranking their relative behaviour and characterising feasibility and limitations in this setting.

### 2.9 Evaluation metrics and statistical analysis

Automated IMRT plans from configurations C1–C6 were quantitatively compared with both the IMRT baseline and the original clinical VMAT plans. The IMRT baseline served as the primary reference on the 61 test cases; clinical VMAT plans were used as an additional clinical benchmark. Evaluation focused on standard RT metrics: target $D_{98\%}$, $D_{2\%}$, $D_{\max}$, and $D_{\text{mean}}$ (all relative to the prescription), the conformity index (CI), and the homogeneity index (HI). CI was computed per Paddick^37^ and HI per ICRU Report 83^38^ (lower HI indicates more homogeneous dose, higher CI indicates better conformity). Fluence maps and field‐dose predictions were also evaluated using voxel‐wise mean squared error (MSE) and mean absolute error (MAE), but these were considered secondary to the plan‐level clinical endpoints.

To assess the impact of MLC sequencing on fluence, we compared predicted fluence maps imported into the TPS with the corresponding fluence after leaf sequencing, using MAE, MSE, and 2D gamma analysis (3%/3 mm, global normalisation, 10% threshold). This quantifies how closely the TPS sequencer preserves the predicted fluence patterns.

Statistical comparisons between automated plans and the IMRT baseline or VMAT plans were performed using paired Wilcoxon signed‐rank tests with Bonferroni‐adjusted significance levels across multiple comparisons. Results are reported as mean ± standard deviation with corresponding p‐values.

### 2.10 Software and hardware

All neural networks were implemented in PyTorch 2.2 (CUDA 12.2) and trained on a workstation with dual NVIDIA RTX A6000 GPUs (48 GB each), which were used in parallel for each training run. Model training times varied by resolution and configuration: 5 mm models (C1–C5) typically converged within 18–30 h, while the 2.5 mm model (C6) required up to 170 h due to increased input dimensionality. Fluence import, leaf sequencing, and final dose calculation were automated via in‐house ESAPI scripts executed in Eclipse v18.0 (Varian Medical Systems) under Windows 10 with ARIA v16.1. Code, trained models, and inference scripts will be made publicly available upon acceptance.

## 3 RESULTS

### 3.1 Clinical baseline: VMAT‐to‐IMRT re‐planning

The automated VMAT‐to‐IMRT re‐planning reproduced the prescription for all 619 cases. Compared with the original VMAT plans, the IMRT baseline had lower hot‐spot indices but compromised on coverage, conformity, and mean dose (Table 3): $D_{2\%}$ −2.17 percentage points (pp) and $D_{\max}$ −2.22 pp (both p<0.001); $D_{\text{mean}}$ −2.41 pp (p<0.001); $D_{98\%}$ −1.66 pp and CI −0.096 (both p<0.001). Overall, the IMRT baseline produced slightly “colder” plans than the clinical VMAT. Plans were evaluated without post‐plan renormalization. The homogeneity index did not differ after Bonferroni correction (p=0.028>α=0.0083). Variability was slightly larger for the IMRT baseline (greater SDs for $D_{2\%}$, $D_{\max}$, $D_{98\%}$, CI).

**TABLE 3:** Clinically delivered VMAT plans versus automatically re‐planned IMRT baseline (619 plans).

Metric	VMAT	IMRT baseline	Δ (VMAT–IMRT)	p
$D_{2\%}$	104.41±1.54	102.24±3.68	+2.17 pp	<0.001
$D_{98\%}$	94.37±2.37	92.71±4.01	+1.66 pp	<0.001
$D_{\max}$	107.97±2.28	105.75±4.13	+2.22 pp	<0.001
$D_{\text{mean}}$	100.23±0.77	97.82±3.39	+2.41 pp	<0.001
CI	0.877±0.055	0.781±0.150	+0.096	<0.001
HI	0.100±0.032	0.097±0.032	+0.003	0.028

Note: Values are mean ± SD; dose metrics are relative to prescription dose. Reported p‐values are from paired Wilcoxon signed‐rank tests with Bonferroni‐adjusted α = 0.0083.

Abbreviations: CI, conformity index; HI, homogeneity index; IMRT, intensity‐modulated radiation therapy; pp, percentage points; SD, standard deviation; VMAT, volumetric modulated arc therapy.

### 3.2 Stage 1: Beam‐wise dose prediction

Table S1 summarizes Stage 1 dose‐prediction performance on the 61‐case test set. Relative to the U‐Net baseline (C1), adding an adversarial discriminator (C2) altered the spatial dose pattern but did not improve target DVH endpoints, and adding the isocenter channel (C3) likewise yielded no clear coverage gain. Pre‐training on multi‐prescription plans (C4) modestly increased coverage ($D_{98\%}$) but left hot‐spot indices largely unchanged.

Introducing the DVH term during fine‐tuning (C5) shifted the target DVHs toward the prescribed clinical criteria, improving all target metrics compared with C1–C4 and achieving higher $D_{98\%}$ and lower $D_{2\%}$ than the IMRT reference (paired Wilcoxon with Bonferroni correction; p<0.001). Increasing the spatial resolution to 2.5 mm (C6) yielded the most favourable DVH trade‐off among the tested configurations, with further gains in coverage over C5 (p<0.001) and the lowest hot‐spot indices. Voxel‐wise MAE is reported in Table S1 for completeness but was not used as an optimization target. Repeating C5 with three independent fine‐tunes produced nearly identical dosimetry ($D_{98\%}=98.0\pm 0.5\%$), indicating negligible stochastic variability at Stage 1.

### 3.3 Stage 2: Fluence mapping and deliverability

**FIGURE 2:**
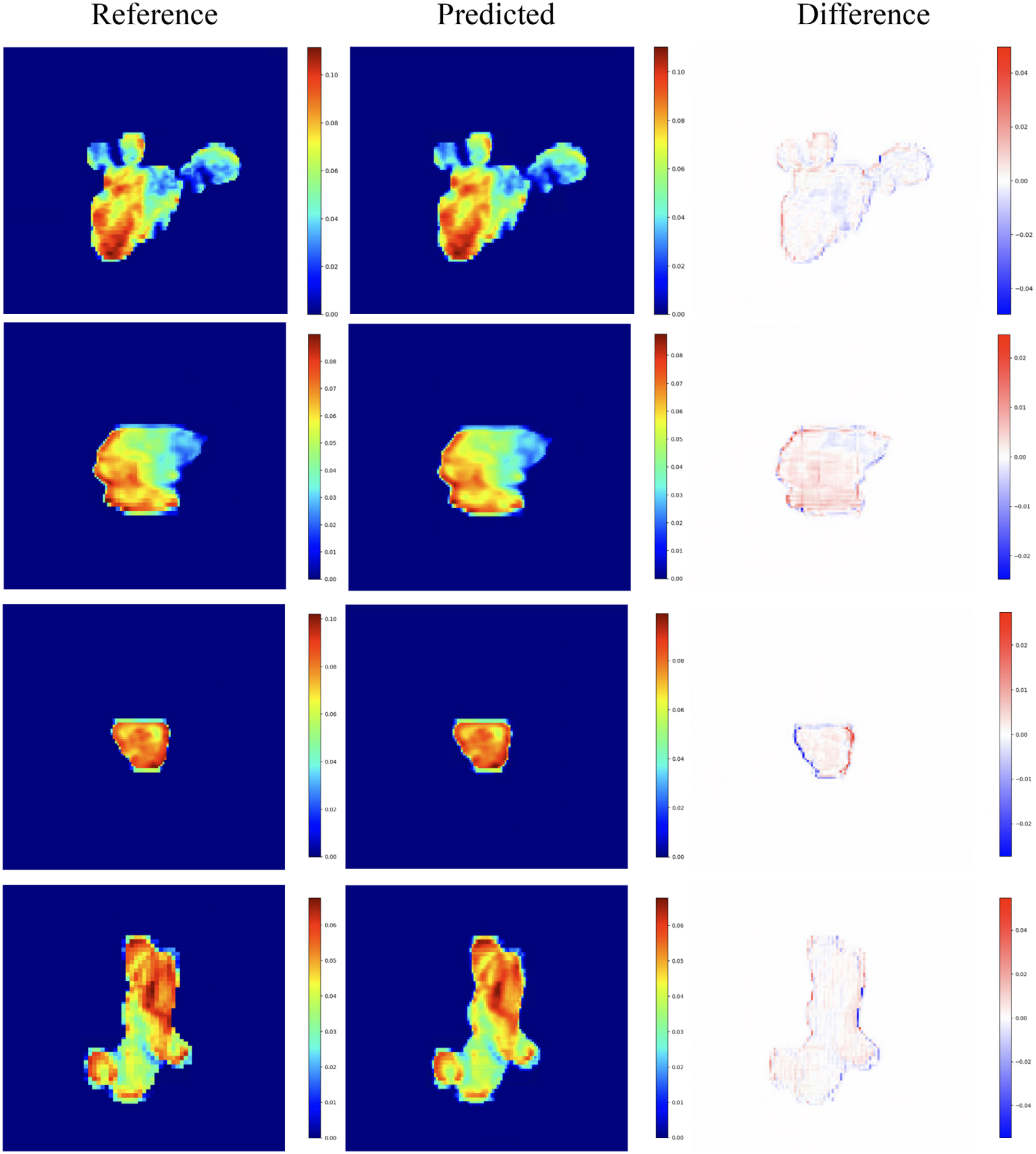
Training‐phase visual assessment of the fluence mapping model. Left: IMRT reference fluence map; middle: predicted fluence; right: absolute difference (|pred−ref|). All maps use the same 128×256 grid and color scale, with values shown as relative intensity. Each row corresponds to a different randomly selected test case and beam angle.

The fluence mapping network was trained as a stand‐alone model to learn the underlying physics for mapping TPS field‐dose volumes to corresponding fluence maps. Low voxel‐wise error was therefore the primary objective of this physics layer. On the held‐out test set, the model achieved MAE = 0.015 ± 0.009 and MSE = 0.013 ± 0.013, with close visual agreement to the reference maps (Figure 2). These results characterise the training‐phase performance of the dose‐to‐fluence mapping.

During inference, the model used the field‐dose predictions from Stage 1 to generate fluence maps. These predicted fluences were imported into Eclipse for MLC leaf sequencing and final dose calculation. The sequenced fluences closely matched the predictions (MAE = 0.0011 ± 0.0006; 2D gamma 95.8 ± 2.6% at 3%/3 mm; Figure S1). This indicates that TPS sequencing introduced only minor local modulations and preserved the beam‐level fluence pattern. No beam failed sequencing, and all deliverable fluences were within clinical modulation limits.

### 3.4 End‐to‐end plan evaluation

**FIGURE 3:**
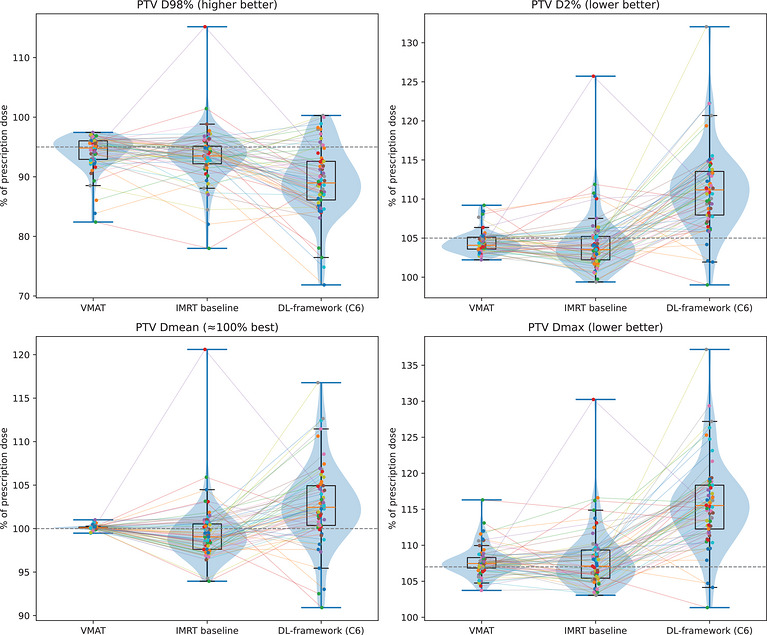
End‐to‐end plan quality for the 61‐case test set comparing clinical VMAT, IMRT baseline, and DL framework for each planning target volume (PTV) metric ($D_{98\%}$, $D_{2\%}$, $D_{\text{mean}}$, $D_{\max}$). Each subplot shows a violin plot with an embedded box plot plus jittered paired lines linking the three techniques for each case, visualizing individual plan‐level differences. Horizontal dashed lines indicate clinical goal thresholds: $D_{98\%}\;\geq 95\%$ (above the line is better), $D_{2\%}\;\leq 102\%$ (below is better), $D_{\max}\;\leq 107\%$ (below is better), and $D_{\text{mean}}\;\approx 100\%$ (closer to the line is better). The DL plans were generated with the C6 configuration, then converted to machine‐deliverable plans by fluence mapping, leaf sequencing, and TPS dose calculation.

**FIGURE 4:**
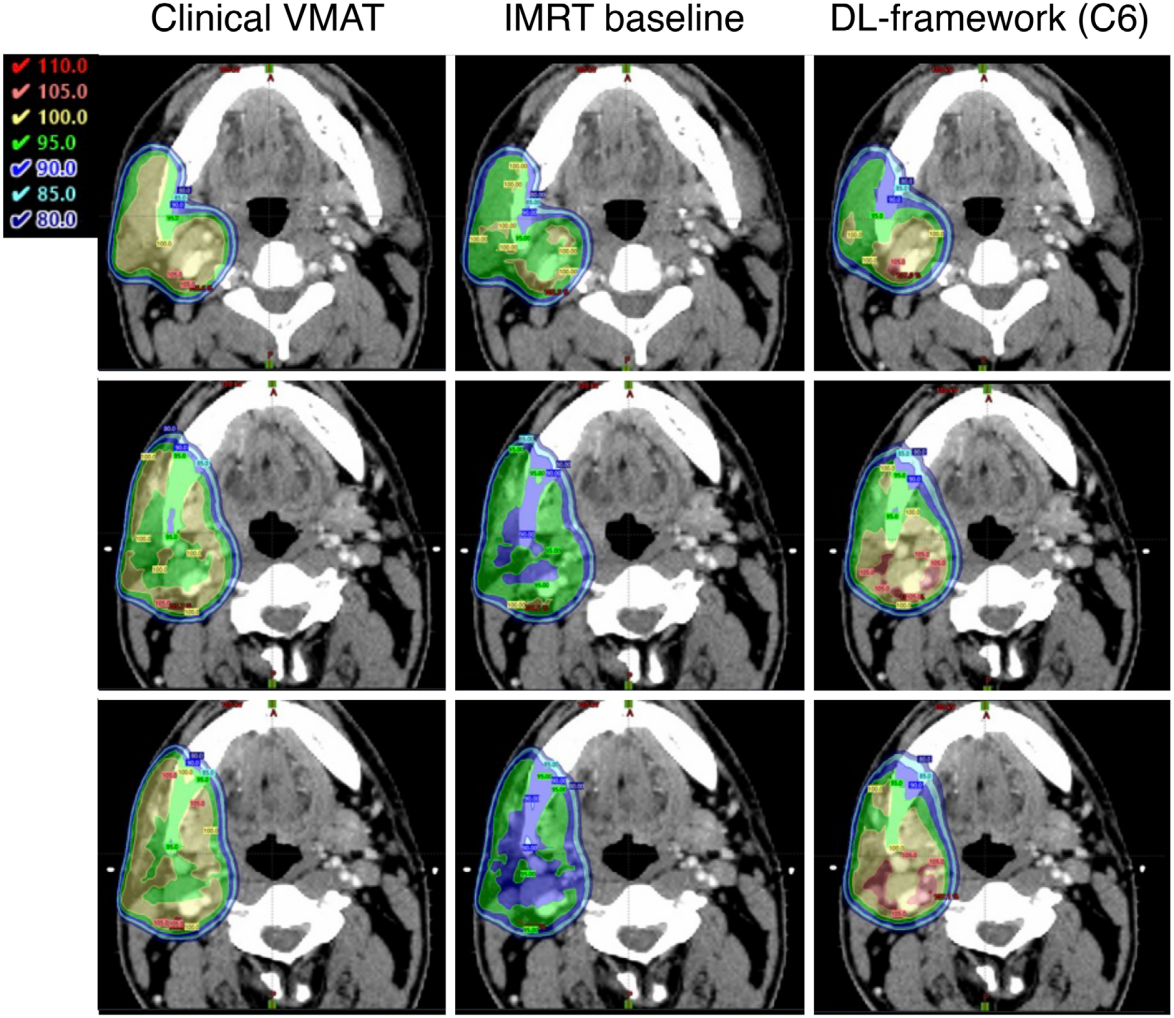
End‐to‐end dose comparison (representative case; curative intent). For three axial positions relative to the isocenter (rows), the clinical VMAT, IMRT baseline, and DL framework (C6) plans are shown (columns). Isodose lines are given as percentages of prescription (e.g., 110%–50%); all panels share the same color scale. (Clinical VMAT dose: AAA v10.0.28; IMRT baseline and DL dose: Acuros XB v18.0.0. IMRT baseline and DL plans were evaluated without post‐plan renormalization; clinical VMAT retains clinical normalization).

Across all configurations, converting Stage 1 dose predictions into fully sequenced IMRT plans (fluence mapping, leaf sequencing, and TPS dose calculation) resulted in a reduction in plan quality compared to the IMRT baseline. For the best‐performing configuration (C6), end‐to‐end plans were significantly worse than the IMRT baseline on all reported target metrics (Figure 3 and Table S2; all p<0.001). A representative case is shown in Figure 4: the clinical VMAT plan encloses most of the PTV within the 100% isodose, the IMRT baseline is slightly less conformal, and C6 shows a retreat of the 98%–100% isodose lines with larger regions covered only by 95%–90%.

**FIGURE 5:**
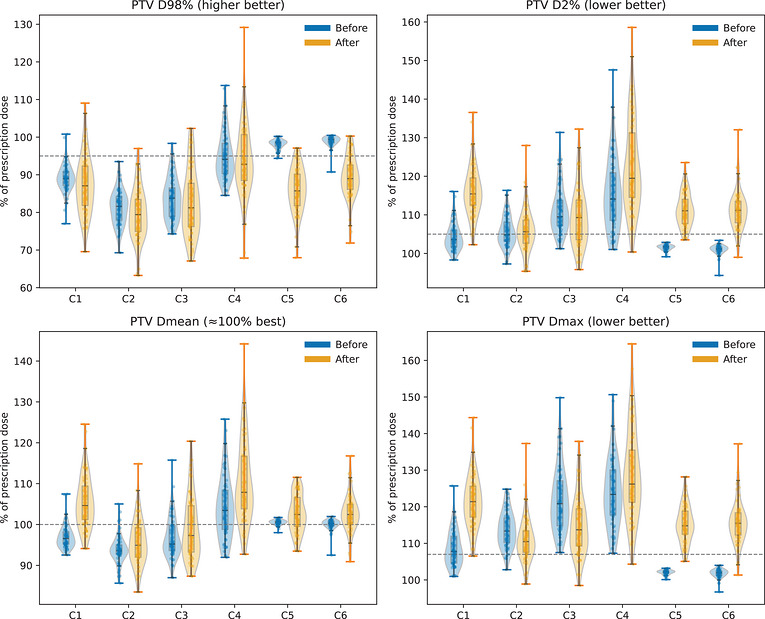
Distributions of planning target volume (PTV) metrics ($D_{98\%}$, $D_{2\%}$, $D_{\text{mean}}$, $D_{\max}$) for all DL configurations: C1; C2 (GAN); C3 (GAN + ISO); C4 (GAN + pre‐train); C5 (C4 + DVH); and C6 (C5 with 2.5mm grid) on the 61‐case test set. Blue violin plots show the Stage 1 DL‐predicted dose, whereas orange violin plots show the final treatment planning system (TPS) dose after Stages 1–3 (DL dose, dose‐to‐fluence conversion, and TPS sequencing), illustrating the DL‐to‐TPS degradation across configurations. Values are expressed as a percentage of the prescribed dose (100%= prescription). Horizontal dashed lines indicate clinical goal thresholds: $D_{98\%}\;\geq 95\%$ (above the line is better), $D_{2\%}\;\leq 102\%$ (below is better), $D_{\max}\;\leq 107\%$ (below is better), and $D_{\text{mean}}\;\approx 100\%$ (closer to the line is better).

The same DL‐to‐TPS degradation pattern was observed for the other configurations (Figure 5 and Table S3), as illustrated by the shift between blue (Stage 1; DL‐predicted dose) and orange (Stages 1–3; DL dose, dose‐to‐fluence, and TPS sequencing) violin plots. C5 and C6, which included the DVH‐based clinical term, produced the most favourable Stage 1 target DVHs among C1–C6 but remained dosimetrically inferior to the IMRT baseline after conversion to machine‐deliverable plans. Notably, even though C2 underperformed C1 at Stage 1, it exhibited a smaller degradation after full TPS sequencing: the summed absolute change across $D_{98\%}$, $D_{2\%}$, $D_{\max}$, and $D_{\text{mean}}$ (∑|Δ|) was significantly lower for C2 than C1 (26.38 vs. 37.77 percentage points; Wilcoxon signed‐rank p=0.00044), suggesting improved end‐to‐end stability for the adversarial configuration.

For comparability, both the IMRT baseline and all DL–generated IMRT plans were evaluated without post‐plan renormalisation (e.g., no rescaling to force target mean to 100% prescription), while clinical VMAT plans retained their clinical normalisation.

## 4 DISCUSSION

In this study, we explored the feasibility and limitations of DL–based end‐to‐end framework for automated IMRT planning in heterogeneous full‐body lymphoma. Configurations that combined a DVH‐based term produced numerically better Stage 1 dose predictions than the IMRT reference, but after fluence mapping and TPS sequencing, all automated plans remained dosimetrically inferior to the IMRT baseline and clinical VMAT. This shows that predicting 3D dose alone does not guarantee the same dose after TPS processing, and may be insufficient for quality assurance,^39^ as it does not fully reflect what is achievable on the treatment machine.

Although the fluence‐mapping model performed well on TPS‐derived field dose–fluence pairs, its role during inference is more challenging. The model was trained to map TPS‐optimized field doses to their corresponding fluence maps, but during inference it operates on generator‐predicted field doses, which may be less physically realistic and/or fall outside the Stage 2 training distribution. This distribution shift may make the learned dose‐to‐fluence mapping less reliable and thereby contribute to the observed degradation after sequencing. Overall, the end‐to‐end degradation likely reflects three linked factors: (i) some Stage 1 field doses fall outside the physically feasible dose space, (ii) the fluence‐mapping model experiences a distribution shift when applied to these unseen dose patterns, and (iii) TPS sequencing and final dose recalculation may further constrain or regularise predicted fluence patterns, despite training on a large cohort of 9285 paired 3D images.^40^

The fluence maps were nevertheless deliverable, as confirmed by TPS sequencing with close agreement between predicted and sequenced fluence (Figure S1). To our knowledge, few FMP studies rigorously examine the effect of MLC sequencing on the predicted fluence patterns.^41^ Although some report deliverable plans, they seldom quantify how the TPS sequencer modifies the model's predicted fluence.^15^, ^24^ Without this check, deliverability can be overestimated: an unrealistic predicted fluence may be regularised by the sequencer, yielding a feasible plan that no longer reflects the model's output.

The behaviour of configurations C1–C6 illustrates how replication, clinical objectives, and end‐to‐end stability interact in this heterogeneous lymphoma setting. Even the replication‐focused variants (C1–C4) that used voxel‐wise MSE to match the IMRT reference struggled at Stage 1, with coverage and hot‐spot metrics already deviating from the reference before any fluence mapping or sequencing, in contrast to studies from more homogeneous single‐site datasets. Adding an adversarial discriminator (C2) changed the spatial dose patterns and reduced the DL–to–TPS degradation compared with the basic supervised U‐Net (C1), while pre‐training on multi‐prescription plans (C4) provided only modest gains in coverage. Configurations C5 and C6, which added a DVH‐based clinical term (and higher resolution in C6), shifted Stage 1 target DVHs toward the prescribed criteria and achieved the most favourable Stage 1 trade‐offs, consistent with the multi‐objective, Pareto nature of inverse planning where strict L1/L2 replication of a single reference can penalise alternative but clinically acceptable solutions. However, after full TPS processing, C5 and C6 still remained dosimetrically inferior to the IMRT baseline, indicating that loss design, regularisation, and dose realism must be considered together, and that improving Stage 1 metrics alone is not sufficient to guarantee clinically competitive plans.

The novelty of this work lies in evaluating DL‐based dose and fluence prediction in a large, clinically heterogeneous lymphoma cohort with TPS integration to assess machine‐deliverability. Lymphoma presents a particularly challenging context for automated planning: targets can arise anywhere in the body, prescriptions span a broad range. Our cohort includes 619 plans from 571 patients, with substantial variability in PTV size, location, and dose prescription. This contrasts with much of the existing literature on dose and fluence prediction, which has focused on anatomically homogeneous sites,^18^, ^42^, ^43^ with standardised prescriptions and organ‐at‐risk sets and often evaluates only network‐predicted dose or fluence without closing the loop through TPS sequencing.^32^, ^44^, ^45^ Comparisons to prior lymphoma work are also limited: for example, vendor knowledge‐based planning models such as RapidPlan have been reported for lymphoma,^10^, ^11^ but these are standardised VMAT models with defined OAR structures, whereas our models were trained on IMRT using CT and PTV only. Because the underlying beam physics is unchanged, we expect the approach to be adaptable to other disease sites with appropriate fine‐tuning, and although we did not optimise explicit OAR objectives or provide OAR contours as inputs, OAR DVHs in randomly selected cases were reasonably close to baseline plans (Figure S2), suggesting some robustness at the planning‐module output.

From a practical perspective, our results do not support immediate clinical use of the proposed framework as an autonomous planning solution. The automated IMRT plans remained dosimetrically inferior to both the IMRT reference and clinical VMAT, and any potential gains in planning time must be weighed against the observed reductions in coverage and increases in hot spots. Nonetheless, the pipeline consistently produced machine‐deliverable plans with target DVHs in the clinical ballpark and required minimal manual interaction, suggesting a possible role as a tool for rapid initial optimisation. The generated first‐pass plans could provide an early estimate of an achievable machine‐deliverable solution, reducing the need to start optimisation from scratch and enabling faster exploration of candidate plans before TPS‐based refinement and clinical review. In addition, the VMAT‐to‐IMRT conversion introduced systematic dosimetric differences, which may have limited the achievable quality of the DL‐generated plans. As this was a feasibility study, a dedicated retrospective plan‐quality QA or filtering step was not applied before model training. Future training on carefully curated, clinically optimised IMRT plans may therefore improve the quality of the final machine‐deliverable predictions. A further limitation is the use of a single‐institution dataset and a single Eclipse/ESAPI‐based workflow; external validation on independent lymphoma cohorts and other centres and TPS workflows is therefore required before generalisability can be established. More broadly, the study illustrates a practical limitation of relying solely on dose prediction. Assessing automated planning methods without explicitly considering dose realism and deliverability could lead to overestimation of clinical performance.

## 5 CONCLUSION

DL–based dose and fluence prediction can generate fast, machine‐deliverable IMRT plans for heterogeneous full‐body lymphoma that lie in the clinical ballpark, but they remain dosimetrically inferior to manually optimised IMRT and VMAT. Even when Stage 1 dose predictions meet or exceed reference DVH criteria, the corresponding end‐to‐end plans after dose‐to‐fluence inversion and TPS sequencing underperform the IMRT baseline, showing that accurate 3D dose prediction alone does not guarantee high‐quality, deliverable plans and that automated planning methods must be evaluated in an end‐to‐end, delivery‐aware manner for complex disease sites such as lymphoma.

## AUTHOR CONTRIBUTIONS

**Denis Kutnár**: Conceptualization; methodology; software; validation; formal analysis; investigation; data curation; visualization; writing—original draft; writing—review and editing; project administration. **Ivan Richter Vogelius**: Supervision; conceptualization; methodology; validation; clinical evaluation; writing—review and editing. **Hristo Atanasov Georgiev**: Investigation; validation; writing—review and editing. **Katrin Elisabet Håkansson**: Methodology; investigation; validation; resources; writing—review and editing. **Lena Specht**: Resources; supervision; validation; writing—review and editing. **Jens Petersen**: Supervision; conceptualization; methodology; software; validation; writing—review and editing; project administration.

## CONFLICT OF INTEREST STATEMENT

The authors declare no conflicts of interest.

## ETHICS STATEMENT

This study was approved by the Ethics Committee of the Capital Region, Copenhagen, Denmark, approval R‐23054402.

## Supporting information



Supporting file

## Data Availability

The data that support the findings of this study are not publicly available due to patient privacy and ethical restrictions. Data may be made available from the corresponding author upon reasonable request and subject to institutional approval.
