# Supplementary material for: End‐to‐end deep learning pipeline for automated IMRT planning in lymphoma: Feasibility and limitations
Source: J Appl Clin Med Phys. 2026 Aug 4;27(8):e70702. doi: 10.1002/acm2.70702 (PMC13438194; doi:10.1002/acm2.70702)
Supplement: Supplementary file 1 [file ACM2-27-e70702-s001.pdf]

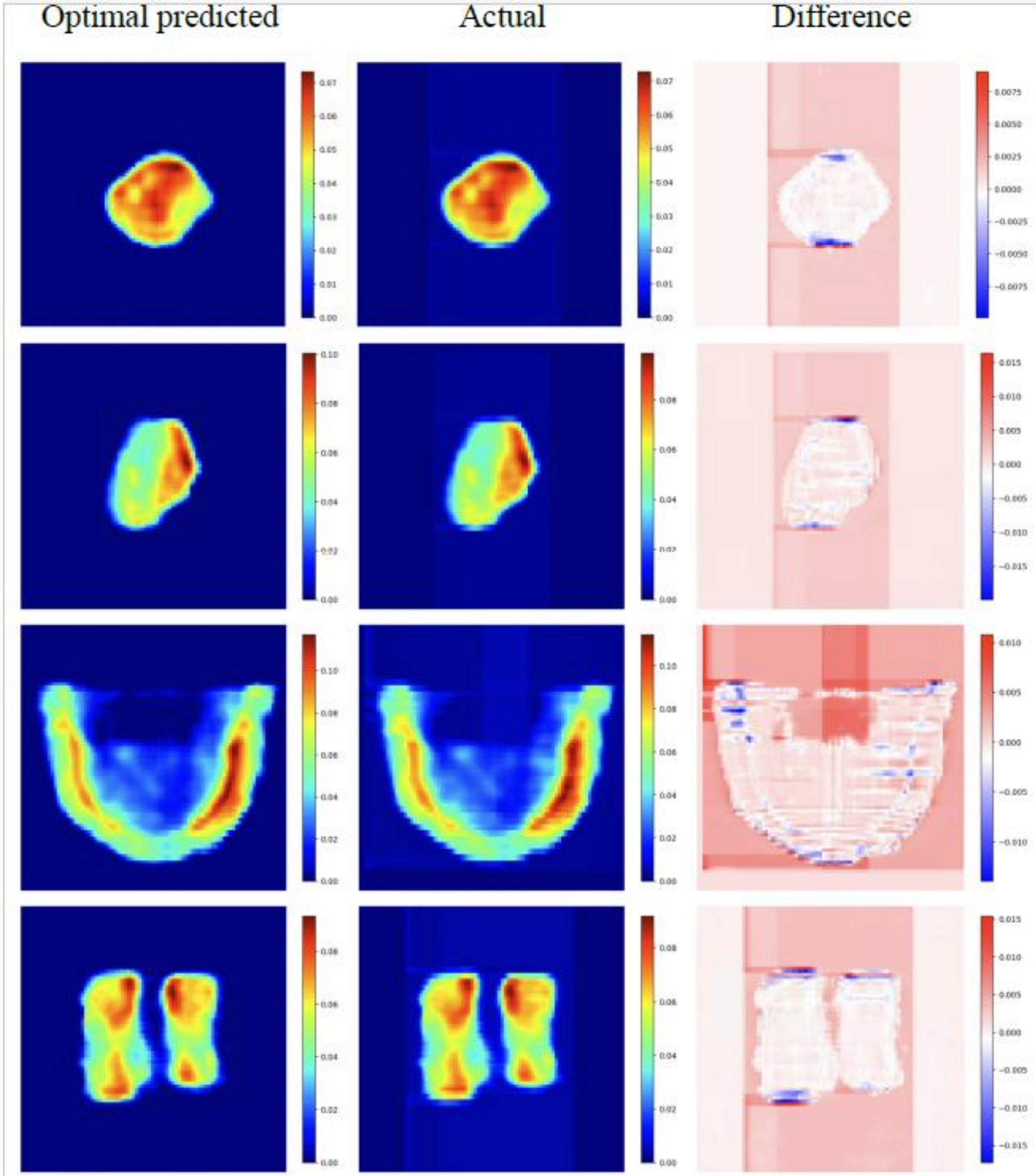

Figure S1. Beam's-eye-view fluence comparison during end-to-end inference. Fluence maps predicted by AutoLymph-IMRT are shown before multileaf collimator sequencing (left), after sequencing in the treatment planning system (middle), and as absolute differences between sequenced and predicted fluence, |sequenced − predicted| (right). Each row corresponds to a randomly selected test case. All maps use the same 128 × 256 grid and colour scale; values are shown as relative intensity.

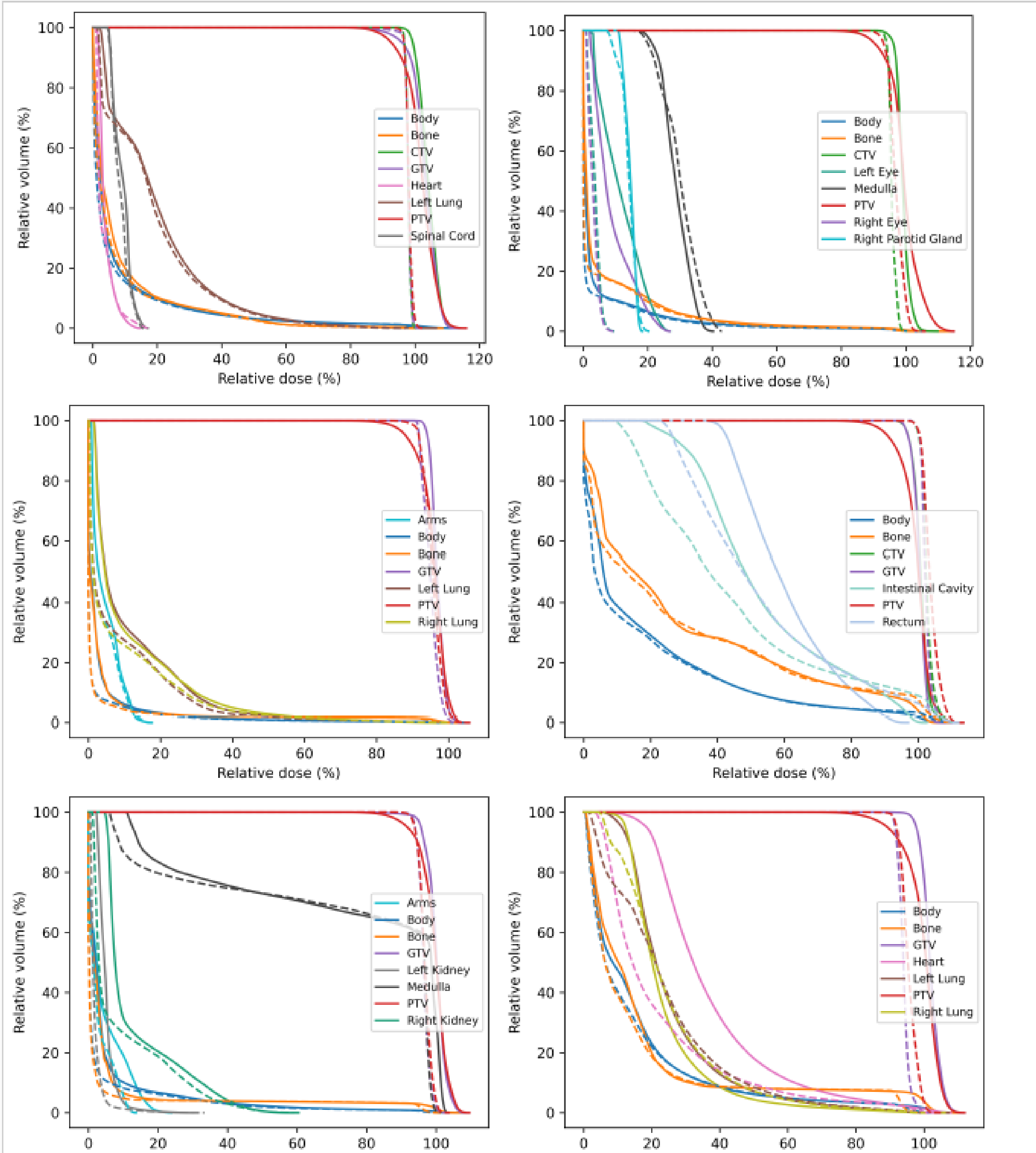

Figure S2. Dose–volume histograms (DVHs) for six randomly selected patients from the test set. Solid curves show the IMRT baseline, and dashed curves show the DL framework using the C6 configuration. Each subplot represents a different treatment plan. Target volumes are labelled as PTV (planning target volume), CTV (clinical target volume), and GTV (gross tumour volume); organs at risk are labelled accordingly in each legend.

Table S1. Dose prediction results (61-case test set)

Dose prediction performance on the 61-case test set. Configurations C1–C6 are compared against the IMRT baseline.

Config	MAE	D98%	D2%	Dmax	Dmean
**IMRT baseline**	—	92.21 ± 6.63	103.72 ± 3.80	105.89 ± 4.05	99.15 ± 3.56
C1	0.31 ± 0.20	89.16 ± 3.79	104.37 ± 3.64	108.47 ± 4.92	97.01 ± 2.91
C2 (GAN)	0.64 ± 0.23	81.32 ± 4.59	105.58 ± 3.92	113.70 ± 4.86	93.91 ± 3.42
C3 (GAN + ISO)	0.48 ± 0.23	83.61 ± 5.50	111.21 ± 6.05	121.94 ± 8.78	96.86 ± 5.00
C4 (GAN + pre-train)	0.49 ± 0.19	95.06 ± 6.44	115.59 ± 9.48	123.95 ± 9.47	104.22 ± 7.64
C5 (C4 + DVH)	0.48 ± 0.23	98.11 ± 1.23†	101.55 ± 0.68†	102.10 ± 0.63	100.51 ± 0.62
C6 (C5, 2.5 mm grid)	0.31 ± 0.17	99.00 ± 1.40‡	100.99 ± 1.25	101.79 ± 1.18	100.04 ± 1.30

Note: Values are mean ± SD. MAE is voxel-wise over the 3D field-dose volumes. D-metrics are expressed as a percentage of the prescription dose. †Bonferroni-corrected paired Wilcoxon test vs. IMRT baseline ($p < 0.001$ for D98% and D2%). ‡Coverage significantly better than C5 ($p < 0.001$).

Abbreviations: DVH, dose–volume histogram; GAN, generative adversarial network; IMRT, intensity-modulated radiation therapy; ISO, isocenter; MAE, mean absolute error; SD, standard deviation.

Table S2. End-to-end plan quality for the 61-case test set

End-to-end plan quality for clinical VMAT plans, the IMRT baseline, and the DL framework. The DL plans were generated with the C6 configuration and converted to deliverable plans by fluence mapping, leaf sequencing, and TPS dose calculation.

Metric	VMAT	IMRT baseline	DL-framework (C6)	p (C6 vs IMRT)
D98%	94.06 ± 3.02	93.52 ± 4.73	89.29 ± 5.82	< 0.001
D2%	104.41 ± 1.36	104.28 ± 3.78	111.18 ± 5.21	< 0.001
Dmax	107.75 ± 2.13	107.94 ± 4.21	115.69 ± 5.95	<0.001
Dmean	100.12 ± 0.24	99.47 ± 3.49	102.72 ± 4.63	<0.001
CI	0.869 ± 0.071	0.852 ± 0.100	0.785 ± 0.117	<0.001
HI	0.103 ± 0.041	0.108 ± 0.037	0.212 ± 0.049	<0.001

Note: Values are mean ± SD. D-metrics are expressed as a percentage of the prescription dose. p-values refer to DL-framework (C6) vs. IMRT baseline and were calculated using paired Wilcoxon signed-rank tests with Bonferroni-adjusted $\alpha = 0.0083$ for six metrics.

Abbreviations: CI, conformity index; HI, homogeneity index; DL, deep learning; IMRT, intensity-modulated radiation therapy; SD, standard deviation; TPS, treatment planning system; VMAT, volumetric modulated arc therapy.

Table S3. End-to-end comparison after TPS sequencing

End-to-end plan quality for all DL configurations after full pipeline processing, including fluence mapping, leaf sequencing, and TPS dose calculation, on the 61-case test set.

Config (after sequencing)	D98%	D2%	Dmax	Dmean
C1	87.35 ± 7.75	116.23 ± 6.48	121.64 ± 7.33	105.57 ± 6.44
C2 (GAN)	79.50 ± 6.87	105.99 ± 5.61	110.98 ± 6.18	95.69 ± 5.62
C3 (GAN + ISO)	82.53 ± 8.70	109.50 ± 7.66	114.76 ± 8.19	98.93 ± 7.57
C4 (GAN + pre-train)	93.62 ± 9.87	122.71 ± 11.90	128.96 ±12.26	110.53 ± 10.0
C5 (C4 + DVH)	85.90 ± 6.09	111.36 ± 4.40	115.27 ± 4.97	102.78 ± 4.61
C6 (C5, 2.5 mm grid)	89.29 ± 5.82	111.18 ± 5.21	115.69 ± 5.95	102.72 ± 4.63

Note: Values are mean ± SD. D-metrics are expressed as a percentage of the prescription dose. Results are shown after full pipeline processing.

Abbreviations: DL, deep learning; DVH, dose–volume histogram; GAN, generative adversarial network; ISO, isocenter; SD, standard deviation; TPS, treatment planning system.
